# Successful Treatment of Severe Paravalvular Leak by Repositioning a Self-Expandable Percutaneous Aortic Valve Bioprosthesis (Evolut PRO+) Using the “Double Snare” Technique

**DOI:** 10.1155/2022/4458109

**Published:** 2022-04-05

**Authors:** Diego H. González-Bravo, Pedro Colón-Hernández, Melanie Quintana-Serrano, Sergio Alegre-Boschetti, Juan Vázquez-Fuster, José J. Acevedo-Valles, Eric Avilés-Rivera

**Affiliations:** ^1^Cardiovascular Division, Department of Medicine, VA Caribbean Healthcare System, San Juan, Puerto Rico, USA; ^2^Cardiovascular Center, Menonita Medical Center, Cayey, Puerto Rico, USA; ^3^Internal Medicine Division, Department of Medicine, VA Caribbean Healthcare System, San Juan, Puerto Rico, USA

## Abstract

Significant (moderate or severe) paravalvular leak (PVL) after transcatheter aortic valve replacement (TAVR) remains a common phenomenon and has been associated with decrease survival and quality of life. Transcatheter valve embolization and migration (TVEM) is a rare post-TAVR complication that can occur in 1% of cases and has been associated with worse patient outcomes. Valve embolization or migration into the left ventricle can result in significant PVL causing hemodynamic instability, shock, heart failure, and hemolytic anemia. Although this complication most commonly occurs in the acute setting (90%) within 4 hours of TAVR, it can also present late (4 hr-43 days later) in 10% of cases. There are no clear guidelines as to how this condition should be managed; however, several percutaneous bailout techniques exist that can ultimately spare the patient from emergent cardiovascular surgery. We present a rare case of late ventricular transcatheter aortic valve migration 3 days after TAVR causing severe PVL and heart failure symptoms that was successfully treated using the percutaneous “double snare” technique.

## 1. Introduction

Transcatheter valve embolization and migration (TVEM) is a dismal complication that can occur in 1% of transcatheter aortic valve replacement (TAVR) cases [1]. It has been associated with increased morbidity and worse patient outcomes. Mortality rates have been reported as high as 40% at 30 days [1, 2]. This complication most commonly presents within the first 4 hours post-TAVR in 90% of TVEM cases; however, the remaining 10% can present late (4 hr-43 days later) for which a high clinical suspicion for diagnosis is imperative [2]. The ascending aorta (38%) is the most common site of valve embolization, followed by the left ventricle (31%) [2]. Among the risk factors for the development of this complication are the use of self-expandable valves, low implantation depths, valve undersizing, large aortic root dimensions, and the presence of a native bicuspid aortic valve [1].

Ventricular migration or embolization of a transcatheter aortic valve can result in significant paravalvular leak (PVL) causing hemodynamic instability, shock, heart failure, and hemolytic anemia [3, 4]. It is well known that post-TAVR significant (moderate or severe) PVL is associated with worse prognosis (17% mortality at 1 year) and poor quality of life [3]. Several percutaneous repair techniques exist that can be employed as “bail-out” procedures often sparing these patients from salvage and emergent cardiac surgery which carries a considerable higher risk of death at 30 days (32.7%) when compared to the percutaneous methods (12.5%) [1]. However, comparative studies to establish superiority between these percutaneous “bail-out” techniques are lacking [4, 5]. Therefore, the selected approach for repair will mostly depend on the PVL mechanism (valve malapposition, undersizing, or malposition) and the capacity of the technique by itself to completely correct the problem as well as the operator's proficiency and familiarity with the selected technique.

We present a rare case of late ventricular TVEM 3 days after TAVR causing severe PVL and heart failure symptoms that was successfully treated using the percutaneous “double snare” technique. We aim to emphasize the importance of early recognition of this complication as we review its diagnosis and highlight its impact over patient morbidity and mortality, plus demonstrate how effective is the “double snare” technique for the treatment of these patients, as well as discuss our rationale behind the selection of this technique.

## 2. Case Presentation

### 2.1. History of Present Illness

A 74-year-old man who presented to our emergency department with symptoms of progressive exertional dyspnea and orthopnea for the last 24 hours. He was found afebrile (37.4°C), hemodynamically stable (heart rate = 81 beats/min and blood pressure = 121/58 mmHg) with a wide pulse pressure (63 mmHg), and tachypneic (22 breaths/min) without hypoxia (96%). Clinical examination revealed signs of central congestion, a grade 3 diastolic decrescendo murmur at the left upper sternal border, and warm extremities without edema. He underwent transcatheter aortic valve replacement (TAVR) 3 days prior at another institution and was discharged home the following day without complications.

### 2.2. Past Medical History

**Figure 1:**
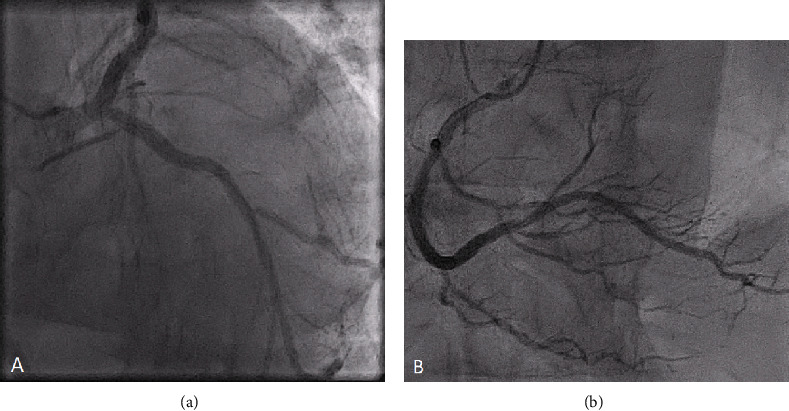
Coronary angiography. No evidence of obstructive coronary artery disease in the left coronary (a) and right coronary arteries (b).

Medical history was significant for mesenteric low-grade follicular B-cell lymphoma, nonobstructive coronary artery disease (Figure 1), and severe aortic valve stenosis with 20-25% left ventricular (LV) systolic function. He underwent TAVR with a 34 mm Medtronic Evolut PRO+ bioprosthesis (supra-annular and self-expanding valve), with plans to proceed with chemotherapy subsequently.

### 2.3. Investigations

**Figure 2:**
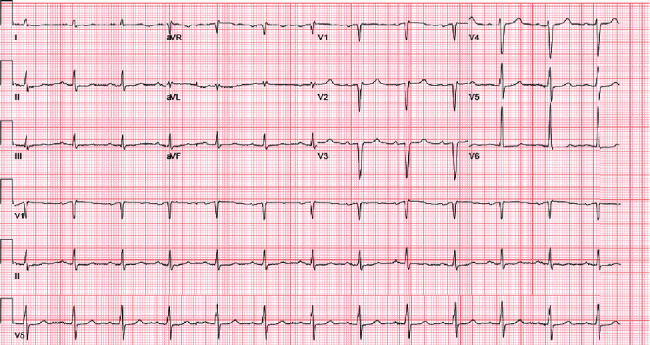
Electrocardiogram. Normal sinus rhythm with old nonspecific ST-segment changes in the lateral leads.

**Figure 3:**
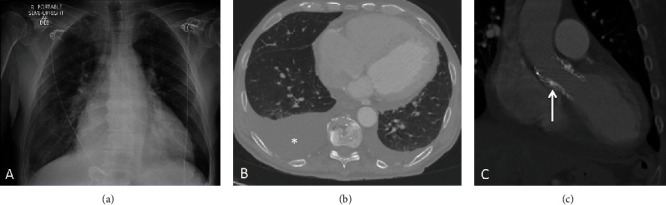
Radiographic studies. (a) Chest X-ray with prominent cephalization of the pulmonary vasculature in favor of pulmonary edema. CT scan showing (b) a moderate right side pleural effusion (asterisk) and (c) low-lying percutaneous aortic valve (arrow).

Electrocardiography revealed sinus rhythm without new ischemic changes (Figure 2). There was no angina or new wall motion abnormalities to favor a type 1 myocardial infarction despite elevated high-sensitive troponins (399 → 362 → 420 ng/L; >22 ng/L abnormal value). No anemia or leukocytosis was present, and pro-BNP was elevated (10,407 pg/mL). Chest X-ray and CT scan (Figure 3) showed a right moderate pleural effusion, pulmonary congestion, and no evidence of pulmonary embolism, aortic dissection, or pericardial effusion.

### 2.4. Differential Diagnosis

Clinical scenario was suggestive of congestive heart failure possibly due to prosthetic valve dysfunction. Nevertheless, given his recent TAVR and hospitalization, pulmonary embolism, aortic dissection, pericardial effusion, pneumonia, anemia, and myocardial infarction were all considered.

### 2.5. Management

**Figure 4:**
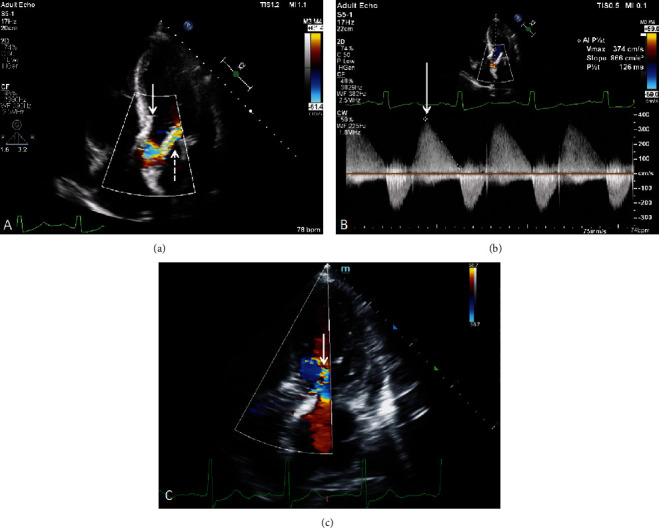
Transthoracic echocardiography. (a) Color Doppler showing left ventricular migration of an aortic bioprosthesis (solid arrow) resulting in PVL (dashed arrow). (b) Spectral Doppler showing a rapidly decelerating (triangle-shaped) aortic regurgitation jet (solid arrow) with a pressure half − time = 126 msec suggestive of severe PVL. (c) Transthoracic echocardiogram 24 hours post-TAVR for comparison. It demonstrates a higher valve position and only mild to moderate PVL when compared to (a).

**Figure 5:**
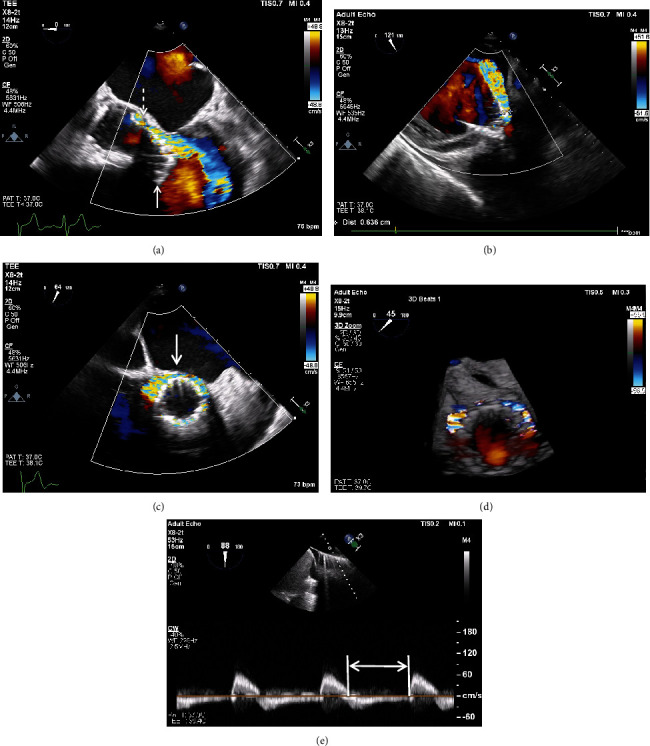
Transesophageal echocardiogram. (a) Aortic bioprosthesis ventricular migration (solid arrow) causing severe PVL (Videos 1–3) by color Doppler (dashed arrow). (b) Transgastric view showing a large vena contracta (0.636 cm). PVL circumferential extent (arrow) involving ~50% of the aortic annulus in the short-axis views in (c) 2D and (d) 3D color Doppler. (e) Thoracic aorta holodiastolic flow reversal consistent with severe PVL (arrow).

Patient was admitted to the intensive care unit, and aggressive diuresis was provided. Transthoracic (TTE) (Figure 4) and transesophageal echocardiogram (TEE) (Figure 5, Videos 1–3) confirmed ventricular transcatheter aortic valve migration causing severe paravalvular leak (PVL) (circumferential extent~50%, aortic holodiastolic flow reversal, large vena contracta (diameter = 0.6 cm, area = 0.57 cm^2^), low pressure half-time (PHT) of 126 msec, and a dilated LV in diastole (7.3 cm)) without evidence of bioprosthesis stenosis (peak systolic velocity (PSV) = 273 cm/sec, acceleration time (AT) = 80 msec, mean pressure gradient (MPG) = 15 mmHg, dimensionless index (DI) = 0.42). Renal, liver, and respiratory systems became compromised (cardiac index = 2.1 L/min/kg and pulmonary wedge pressure = 28 mmHg) requiring dobutamine and noninvasive ventilation for stabilization. Case was presented to patient's TAVR operator who recommended transferring the patient to his institution for PVL intervention.

**Figure 6:**
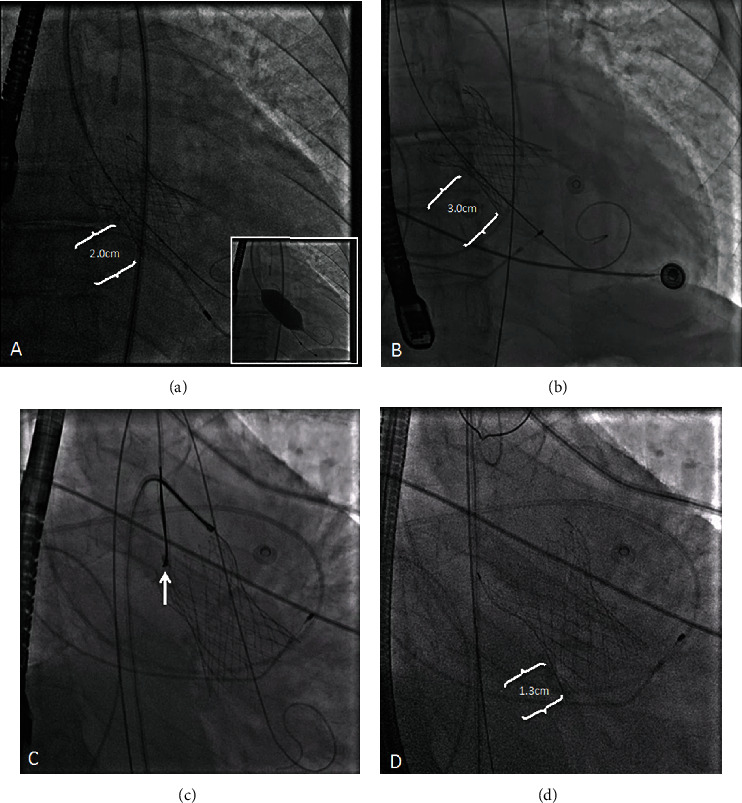
“Double snare” technique (fluoroscopy). (a) Low-lying Evolut PRO+ after initial implantation and postdilation (inset) with its inflow 2 cm below the aortic annulus (braces) for comparison. (b) Late ventricular valve migration and embolization into the left ventricle as the distance from the aortic annulus to the valve inflow worsened (3.0 cm) resulting in severe paravalvular leak. (c) The inner loop tab of the valve was snared first (arrow) via the right radial artery followed by the outer loop tab via the right femoral artery. Both snares were pulled to reposition the valve cranially. (d) After repositioning, the aortic annulus-to-valve inflow distance (braces, 1.3 cm) decreased and PVL improved.

**Figure 7:**
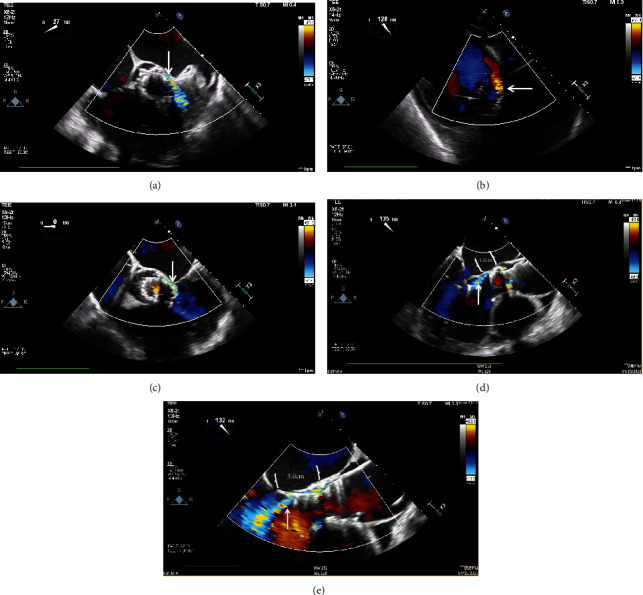
Transesophageal echocardiogram after valve repositioning. Mild PVL by color Doppler (arrow) in the (a) short-axis and (b) deep-transgastric views (Videos 4 and 5). (c) PVL circumferential extent (arrow) was reduced to 20%. (d) Long-axis view showing mild PVL (arrow). (e) Severe PVL (arrow) prior to valve repositioning for comparison. Postrepositioning, the aortic annulus-to-valve inflow distance (braces) was reduced from (e) 3.0 cm to (d) 1.3 cm.

Via right radial and femoral arterial approach (6Fr), two Medtronic Amplatz Goose Neck Microsnares (15 × 120 mm) were advanced into the ascending aorta using two AL1 catheters. The inner loop tab of the valve was snared first followed by the outer loop tab (Figure 6). The snares were then pulled constantly for 15-20 sec, and this was repeated every 20 sec until real-time TEE showed valve repositioning to a higher level resulting in improvement of PVL from severe to mild (Figure 7, Videos 4 and 5). No postdilation was necessary after valve repositioning as described similarly in other reports [6–8]. Additionally, rapid ventricular pacing was not required nor performed during valve repositioning.

## 3. Discussion

**Figure 8:**
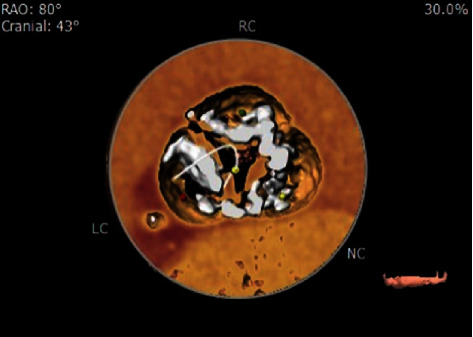
Extensive aortic valve calcifications (white). Right (RC), left (LC), and noncoronary (NC) cusps.

Late TVEM and severe PVL were precipitated in our patient by several factors. First, upfront balloon predilation to ensure optimal bioprosthesis expansion without migration was not performed because the risk of stroke was believed to be unacceptably high given the severely complex calcific aortic valve (Figure 8). Second, valve oversizing > 10% has been shown to decrease the risk of future PVL; however, it could not be achieved in our case despite using the largest (34 mm) bioprosthesis available given that our patient's aortic annulus was relatively larger (CT measurements: area = 771.4 mm^2^, diameter = 31.8 mm, perimeter = 99.9 mm) [4]. Both factors precipitated moderate PVL immediately post-TAVR due to valve malapposition and a low malposition (Figure 6(a)) despite our intentions to implant the valve in the supra-annular position with commissural alignment. Valve repositioning prior to full deployment could not be performed as the patient was poorly tolerating the procedure hemodynamically given his severely decreased LV systolic function. Consequently, the residual moderate PVL forced us to perform balloon postdilation (BPD) with a Z-Med 30 × 40 mm valvuloplasty balloon resulting in the improvement of the PVL to a mild-to-moderate degree (Figure 4(c)). We accepted this result and opted to not be more aggressive with BPD as it has been shown to increase risk of stroke, leaflet injury, aortic rupture, and pacemaker implantation [4].

The relative size mismatch, lack of predilation, low malposition, and failure to perform a more aggressive BPD favored a “watermelon seeding” phenomenon that made the self-expanding valve slip into the LV resulting in late ventricular TVEM and severe PVL 3 days later after TAVR. At this stage, vascular plugs were not an option given the incorrect valve position, circumferential nature of the PVL, and its multiple jets [5]. Performing valve-in-valve TAVR was contemplated [4, 5]. Nevertheless, the transcatheter bioprosthesis was so deep in the LV that the sealing skirt of a second valve was going to be incapable of sealing the aortic annulus and the entire adjacent tissueless valve frame that was allowing blood to percolate back to the LV, much less overcome its radial strength. Furthermore, performing valve-in-valve TAVR has been shown to increase the risk of pacemaker implantation, acute coronary artery occlusion, may compromise future coronary access, and can indeed increase procedure cost [6]. Alternatively, the snare technique could bypass much of these challenges and correct the PVL effectively.

Vavouranakis et al. [6] were the first one to describe the snare technique by using a single Amplatz Goose Neck Microsnare (35 × 120 mm) to successfully reposition a low-implanted Medtronic CoreValve and resolve its PVL. This procedure is not to be confused with the snare technique used for facilitating advancement of the TAVR delivery system across challenging aortic anatomy and the aortic valve [9]. Adding a second snare as we did in this case, a maneuver known as the “double snare” technique, has also been described as a way to augment the pulling force and likelihood of successful repositioning [7]. We used the right radial approach to snare the contralateral tab of the valve at the inner aortic curve and the femoral approach to snare from the opposite side the remaining valve tab at the outer aortic curve. By using this configuration of contralateral forces crossing each other, the radial force of the valve was decreased as it was being forced to partially collapse on itself which in turn resulted in less friction and tissue damage as the valve was being pulled. Nonetheless, awareness should be made that aggressive pulling of the bioprosthesis could result in TVEM into the ascending aorta, embolic stroke, or aortic dissection [6–8]. Despite all these risks, a retrospective study by Won-Keun et al. [1] showed that the use of this percutaneous bailout strategy was found to have a lower 30-day mortality of 12.5% compared to performing a transcatheter valve-in-valve procedure (16.3%) and open-heart surgery (32.7%).

Postdilation after valve repositioning using the “double snare” technique was not performed for several reasons. First, we wanted to minimize further aggressive instrumentation of the ascending aorta knowing how severely calcific and friable the native aortic valve was, in order to decrease our risk of causing embolic complications, especially in our setting where we do not have available the cerebral embolic protection system (SENTINEL, Boston Scientific). Second, we believed that the risks (e.g., permanent pacemaker implantation, aortic rupture, and bioprothesis leaflet injury) were greater than the benefits, as we had used the largest diameter valvuloplasty balloon (30 × 40 mm, Z-MED) that we had available for postdilation after initial valve implantation, and it was unsuccessful in preventing late ventricular migration of the valve. And third, several similar reports using the “double snare” technique have demonstrated successful results without performing postdilation after valve repositioning [6–8].

The snare technique usefulness has been mostly described as a bailout procedure for the treatment of low valve implantation or acute (<1-4 hr) TVEM resulting in significant PVL. Our case is unique and exceptional since it shows that this technique can also be safe and effective for the treatment of late ventricular TVEM even when occurring days later after TAVR, as it was done successfully in our patient on day 7 post-TAVR sparing him from emergent open-heart surgery.

## 4. Follow-Up

After valve repositioning, patient's symptoms resolved, renal and liver function normalized, and discontinuation of dobutamine and noninvasive ventilation was achieved. Permanent pacemaker implantation was not required. Subsequent TTE confirmed mild PVL (PHT = 490 msec) without patient-prosthesis mismatch or stenosis (PSV = 151 cm/sec, MPG = 5 mmHg, AT = 70 msec, DI = 0.62, indexed effective orifice area = 1.47 cm^2^/m^2^). Cardiac rehabilitation was provided and discharged home on heart failure guideline-directed medical therapy with oncology follow-up for chemotherapy coordination.

## 5. Conclusion

Transcatheter valve embolization and migration (TVEM) and significant PVL post-TAVR are associated with worse patient outcomes, poor quality of life, and increased mortality. The “double snare” technique is a useful bailout procedure for the treatment of significant PVL post-TAVR due to low valve malposition and acute TVEM. However, this case is unique as it demonstrates that this technique may also be safe and effective for the treatment of late ventricular TVEM causing significant PVL.

## Data Availability

The data and test results used in this case report are all included in this article.

## Abbreviations

AT: Acceleration time
BPD: Balloon post-dilation
DI: Dimensionless index
MPG: Mean pressure gradient
LV: Left ventricle
PSV: Peak systolic velocity
PHT: Pressure half-time
PVL: Paravalvular leak
TAVR: Transcatheter aortic valve replacement
TEE: Transesophageal echocardiogram
TTE: Transthoracic echocardiogram
TVEM: Transcatheter valve embolization and migration.

## Supplementary Materials

Supplementary Materials The transesophageal echocardiogram videos are shown in https://drive.google.com/drive/folders/1huM-u0rMZXKcDuiqL0_ebIHZGE1T1Hst?usp=sharing. Video 1: Five-chamber view severe PVL pre-repositioning. Video 2: Deep transgastric view severe PVL pre-repositioning. Video 3: Short-axis view severe PVL pre-repositioning. Video 4: Long-axis view mild PVL post-repositioning. Video 5: Deep transgastric view mild PVL post-repositioning.
