# Supplementary material for: Successful Treatment of Severe Paravalvular Leak by Repositioning a Self-Expandable Percutaneous Aortic Valve Bioprosthesis (Evolut PRO+) Using the “Double Snare” Technique
Source: Case Rep Cardiol. 2022 Apr 5;2022:4458109. doi: 10.1155/2022/4458109 (PMC9005318; doi:10.1155/2022/4458109)
Supplement: Supplementary Materials — The transesophageal echocardiogram videos are shown in https://drive.google.com/drive/folders/1huM-u0rMZXKcDuiqL0_ebIHZGE1T1Hst?usp=sharing. Video 1: Five-chamber view severe PVL pre-repositioning. Video 2: Deep transgastric view severe PVL pre-repositioning. Video 3: Short-axis view severe PVL pre-repositioning. Video 4: Long-axis view mild PVL post-repositioning. Video 5: Deep transgastric view mild PVL post-repositioning. [file 4458109.f1.zip › Video Legends for supplementary material description.docx]

**Title: Successful treatment of severe paravalvular leak by repositioning a self-expandable percutaneous aortic valve bioprosthesis (Evolut PRO+) using the “double snare” technique**

Diego H. González-Bravo, MD^1 (MD)^; Pedro Colón-Hernández, MD^3 (MD)^; Melanie Quintana-Serrano, MD^1 (MD)^; Sergio Alegre-Boschetti, MD^1 (MD)^; Juan Vázquez-Fuster, MD^1 (MD)^; José J. Acevedo-Valles, MD^1,2 (MD)^; and Eric Avilés-Rivera, MD^1,3 (MD)^

^1^Cardiovascular Division, Department of Medicine,

VA Caribbean Healthcare System, San Juan, Puerto Rico.

^2^Internal Medicine Division, Department of Medicine,

VA Caribbean Healthcare System, San Juan, Puerto Rico.

^3^Cardiovascular Center, Menonita Medical Center, Cayey, Puerto Rico.

**Video Legends** (<https://drive.google.com/drive/folders/1huM-u0rMZXKcDuiqL0_ebIHZGE1T1Hst?usp=sharing>)

[**Video 1:**](https://drive.google.com/file/d/1BjZm9s-stkSAOdtA5o6yoxxqkWrebAmf/view?usp=sharing) Transesophageal echocardiogram mid-esophageal 5-chamber view prior to valve repositioning showing severe paravalvular leak due to late ventricular transcatheter valve embolization and migration. Ventricular migration and embolization of the Evolut PRO+ bioprosthesis is causing impingement of the anterior mitral valve leaflet resulting in its restrictive opening. The low-lying bioprosthesis is causing severe paravalvular leak by color Doppler that is posteriorly directed and traveling back to the ventricle during diastole between the bioprosthesis and the aorto-mitral curtain.

[**Video 2:**](https://drive.google.com/file/d/14iM27FtKxo-JbzcN2P6x2OjRewhfH5NX/view?usp=sharing) Transesophageal echocardiogram deep trans-gastric view with color Doppler showing severe paravalular leak prior to valve repositioning. Paravalvular regurgitation jet has a large vena contracta (diameter=0.6cm, area=0.57cm^2^) which is suggestive of a severe leak. A dilated and dysfunctional left ventricle can also be appreciated.

[**Video 3:**](https://drive.google.com/file/d/1sbXZ4awJapS8Zc-7LTl0h9vMrOxQQ17m/view?usp=sharing) Transesophageal echocardiogram mid-esophageal short-axis view showing severe paravalvular leak before valve repositioning using the “double snare” technique. Paravalvular leak involves approximately 50% of the bioprosthesis circumference which favors the presence of severe regurgitation.

[**Video 4:**](https://drive.google.com/file/d/11mpCdT5qPbij7AN7TNDP2ZEQu2eng84C/view?usp=sharing) Transesophageal echocardiogram mid-esophageal long-axis view showing interval improvement of severe paravalvular leak to a mild degree after valve repositioning using the “double snare” technique. Evolut PRO+ bioprosthesis is not further causing impingement against the anterior mitral valve leaflet after being repositioned to a higher level. Mild functional mitral regurgitation is present.

[**Video 5:**](https://drive.google.com/file/d/1keBgJjmHGkj5yyrMbgyCqHwlVok-LVjY/view?usp=sharing) Transesophageal echocardiogram deep trans-gastric view with color Doppler showing improvement of severe paravalvular leak to a mild degree after successful valve repositioning. Pressure half-time of the severe paravalvular leak improved from 126 to 490msec in favor of a residual mild leak.
